# Effects of dimethyl fumarate on neuroprotection and immunomodulation

**DOI:** 10.1186/1742-2094-9-163

**Published:** 2012-07-07

**Authors:** Philipp Albrecht, Imane Bouchachia, Norbert Goebels, Nadine Henke, Harald H Hofstetter, Andrea Issberner, Zsuzsa Kovacs, Jan Lewerenz, Dmitrij Lisak, Pamela Maher, Anne-Kathrin Mausberg, Kim Quasthoff, Corinna Zimmermann, Hans-Peter Hartung, Axel Methner

**Affiliations:** 1 Department of Neurology, Heinrich-Heine-University Düsseldorf, Medical Faculty, Düsseldorf, 40225, Germany; 2 Department of Neurology, University Hospital of Ulm, Ulm, 89081, Germany; 3 Cellular Neurobiology Laboratory, Salk Institute for Biological Studies, La Jolla, CA, 92037, USA; 4 Neurologische Klinik der Heinrich-Heine-Universität Düsseldorf, Moorenstr. 5, D-40225, Düsseldorf, 40225, Germany

**Keywords:** Dimethyl fumarate, Oxidative stress, Neuroprotection, Neuromodulation

## Abstract

**Background:**

Neuronal degeneration in multiple sclerosis has been linked to oxidative stress. Dimethyl fumarate is a promising novel oral therapeutic option shown to reduce disease activity and progression in patients with relapsing-remitting multiple sclerosis. These effects are presumed to originate from a combination of immunomodulatory and neuroprotective mechanisms. We aimed to clarify whether neuroprotective concentrations of dimethyl fumarate have immunomodulatory effects.

**Findings:**

We determined time- and concentration-dependent effects of dimethyl fumarate and its metabolite monomethyl fumarate on viability in a model of endogenous neuronal oxidative stress and clarified the mechanism of action by quantitating cellular glutathione content and recycling, nuclear translocation of transcription factors, and the expression of antioxidant genes. We compared this with changes in the cytokine profiles released by stimulated splenocytes measured by ELISPOT technology and analyzed the interactions between neuronal and immune cells and neuronal function and viability in cell death assays and multi-electrode arrays. Our observations show that dimethyl fumarate causes short-lived oxidative stress, which leads to increased levels and nuclear localization of the transcription factor nuclear factor erythroid 2-related factor 2 and a subsequent increase in glutathione synthesis and recycling in neuronal cells. Concentrations that were cytoprotective in neuronal cells had no negative effects on viability of splenocytes but suppressed the production of proinflammatory cytokines in cultures from C57BL/6 and SJL mice and had no effects on neuronal activity in multi-electrode arrays.

**Conclusions:**

These results suggest that immunomodulatory concentrations of dimethyl fumarate can reduce oxidative stress without altering neuronal network activity.

## Background

Chronic disability in multiple sclerosis (MS) is due to neuronal degeneration, which is not amenable, or is incompletely amenable to immunomodulatory therapy. The mechanisms remain elusive, but there is accumulating evidence that oxidative stress may play a key role [1-3]. Dimethyl fumarate (DMF) is a novel oral therapeutic agent which reduces disease activity and progression in patients with relapsing-remitting MS [4,5]. Previously suggested immunomodulatory mechanisms of action of DMF or its metabolite monomethyl fumarate (MMF) include inhibition of cytokine-induced nuclear translocation of the nuclear factor kappa B (NF-κB) [6], apoptosis of stimulated T cells [7], and increased production of the TH2 cytokines IL-4 and IL-5 in stimulated T cells, whereas generation of the TH1 cytokine interferon gamma (IFN-γ) [8] is supposed to remain unaffected. DMF also activates the transcription factor Nrf2 (nuclear factor erythroid 2-related factor 2), which binds to antioxidant response elements in the promoters of protective genes such as NADPH-quinone-oxidoreductase-1 (NQO1) [9] and heme-oxygenase-1 [10]. This ultimately raises the levels of the important intracellular antioxidant glutathione [9,10]. However, short-term incubation with DMF for 60 minutes decreases the glutathione content of cortical primary cultures and OLN-93 cells [11,12].

Here, we first investigated the concentration and time dependence of DMF-mediated protection in neuronal cells using a model of endogenous oxidative stress, oxidative glutamate toxicity, where extracellular glutamate blocks the glutamate-cystine antiporter system Χc-. This leads to deprivation of cystine and its reduced form cysteine, the rate-limiting substrate for the synthesis of glutathione. The subsequent glutathione depletion gives rise to the accumulation of reactive oxygen species and cell death by oxidative stress (recently reviewed [13]). We show herein that neuroprotective concentrations of DMF suppress cytokine production by splenocytes from two different mouse strains without effecting apoptosis and do not impact neuronal network activity studied with dissociated cortical cultures grown on multi-electrode arrays [14] which allows a highly sensitive and reproducible assessment of network activity. Our results suggest that low doses of DMF may promote cellular resistance against oxidative stress and cause immunomodulation independent of T cell apoptosis or alterations in endogenous brain activity.

## Materials and methods

### Material

DMF and MMF (sodium salt) for all experiments were obtained from Biogen Idec, Carl-Zeiss-Ring 6 85737 Ismaning, Germany and solubilized in dimethylsulfoxide (DMSO), which was also used as the vehicle control. The pH of all media was kept constant at 7.4. Cell culture dishes were from *Greiner Bio*-*One*, Maybachstraße 2., 72636 Frickenhausen, Germany; DMEM cell culture medium, sterile phosphate buffered saline were from PAA, Unterm Bornrain 2, 35091 Cölbe, Germany; penicillin, streptomycin were from Gibco/Life Technologies, Frankfurter Straße 129B, 64293 Darmstadt, Germany; cryopreserved primary dissociated cortical cultures from embryonic rats were from QBM Cell Science Inc., 1200 Montreal Road, Building M23A, Suite 147, Ottawa, Ontario, Canada; Cell Titer Blue was from Promega, Schildkrötstraße 15 68199 Mannheim, Germany; the high contact imaging microscope, the anti-CD3 antibody, 7-AAD and Annexin V PE were from Becton Dickinson, Tullastr. 8-12, 69126 Heidelberg, Germany; the anti-Nrf2 antibody was from Santa Cruz Biotechnology, Bergheimer Straße 89, 69115 Heidelberg, Germany; the anti-Actin antibody and secondary antibodies were from Millipore, 290 Concord Road Billerica, MA 01821, USA; the anti-NF-κB-antibody was from Cell Signaling Technologies, 3 Trask Lane Danvers, MA 01923, USA; multi-electrode arrays were from Multichannel Systems, Aspenhaustrasse 21. 72770 Reutlingen*,* Germany; the MEA analyzing software Spanner was from RESULT software, 47918 Tönisvorst, Germany; the Universal Probe Library^TM^ was from Roche, Emil-Barell-Str. 1 79639 Grenzach-Wyhlen, Germany; Fam-Tamra labeled oligonucleotides were from Eurofins-MWG-Operon, Anzingerstr. 7a, 85560 Ebersberg, Germany; the TNFα ELISA was from R&D Systems, Borsigstrasse 7. 65205 Wiesbaden, Germany; the Immunospot Analyzer was from CTL, 2860 Fisher Road, Columbus, OH 43204, USA; Prism software was from GraphPad Software, 2236 Avenida de la Playa, La Jolla, CA 92037, USA; spreadsheet software was from Microsoft, Konrad-Zuse-Str. 1, 85716 Unterschleißheim, Germany; all other chemicals were from Sigma Aldrich, Georg-Heyken-Str. 14 D-21147 Hamburg Germany.

### Cell culture, viability assays and glutathione measurement

The preparation of embryonic primary cortical cultures and splenocyte cultures from C57BL/6 and SJL mice and the cell culture of HT22 and fibroblast cells were performed as described [15,16]. For the analysis of network activity, cryopreserved primary dissociated cortical cultures from embryonic rats (embryonic day 18, E18, QBM Cell Science) were employed. After thawing, the cells were plated at a final density of 10^5^ cells on PDL-/laminin-coated multi-electrode arrays (MEAs) or coverslips. Neuronal cultures were incubated in a humidified atmosphere (5% CO_2_/95% air) at 37 °C for 24 h in DMF or vehicle prior to glutamate treatment. Viability was quantitated 24 h after glutamate addition by the Cell Titer Blue (CTB) assay (Promega) and normalized to vehicle treatment. Total glutathione was measured enzymatically as described previously [15] and normalized to cellular protein measured by the bicinchoninic acid-based method (Pierce). Glutathione released into the cell culture medium was also quantitated enzymatically after 4 h in cystine-free medium and normalized to total cellular protein. Cell viability of splenocytes was assessed using flow cytometry quantitating 7-AAD (BD Pharmingen #51-68981E) and Annexin V PE (BD Pharmingen #556421) stained cells according to manufacturers’ protocols.

### Cell fractionation, SDS-PAGE and immunoblotting

Differential detergent fractionation and immunoblotting were performed as previously described [15] using anti-Nrf2 (1:1000; Santa Cruz Biotechnology; #SC13032) and anti-Actin (1:3000; Millipore, MAB1501) antibodies.

### Translocation analysis of NF-κB and Nrf2

Intracellular localization of transcription factors was quantitated by high-content imaging using a BD Pathway 855 microscope (BD Biosciences). HT22 cells were fixed with 4% paraformaldehyde and permeabilized with 0.3% Triton-X 100, blocked with Roti ImmunoBlock (Roth #144.1) for 1 h before they were incubated with primary antibodies (NF-κB: Cell Signaling #9936 S, Nrf2: Santa Cruz #sc-13032) overnight and stained with secondary fluorochrome-labeled antibodies (Millipore #AP132F and #124 F). Fluorescence intensities in regions of interest defined by the nuclear stain DAPI (150 nM) were compared with those of a second concentric band surrounding the nuclei and corresponding to the cytoplasm.

### Quantitative real-time PCR

RNA extraction, reverse transcription and quantitative real-time PCR were performed as previously described [15] using Fam/Dark-quencher probes from the Universal Probe Library^TM^ (Roche) or individually designed Fam/Tamra probes (MWG). Beta-actin and HPRT served as endogenous control genes and showed no differential expression after incubation with DMF. Primer and probe sequences can be obtained from the authors.

### TNFα ELISA

Primary splenocytes from 6- to 8-week old female C57BL/6 and SJL mice were stimulated with 1 μg/ml anti-CD3 (BD Bioscience) and treated with 1, 10 and 100 μM of DMF. Supernatants were collected after 48 h and concentration of TNFα was measured following the manufacturer’s protocol (R&D Systems).

### Cytokine enzyme-linked immunosorbent spot (ELISPOT) assays

ELISPOT assays were essentially performed as previously described [16] using splenocytes from 6- to 8-week old female C57BL/6 and SJL mice. Splenocytes were incubated with 10 μM DMF or vehicle for 24 h while stimulated with 0.5 μg/ml anti-mouse CD3. Computerized ELISPOT analysis was done using an Immunospot Analyzer (CTL).

### Extracellular microelectrode recordings and signal analysis

Extracellular microelectrode recordings and signal analysis were performed as described [14]. Network activity was recorded on multi-electrode arrays (MEAs) (Multi Channel Systems) with 64 titanium nitride electrodes (30 μm diameter and 200 μm spacing) at 37 °C using sterile conditions. Signals from all 64 electrodes were simultaneously sampled at 25 kHz, visualized and stored using the standard software MC-Rack (Multi Channel Systems). Spike and burst detection was performed offline using specialized software (SPANNER 2.0, Result, Germany).

### Statistical analysis

Statistical analysis was performed using spreadsheet (Microsoft Excel) and Prism (Graphpad) software. Multiple group analyses were conducted using two-way analysis of variance (ANOVA) and Bonferroni or Dunnett’s post hoc test, and comparison of two groups using the two-tailed *t*-test. *P*-values < 0.05 were considered significant.

## Results

### DMF protects cells from oxidative stress by enhancing Nrf2 abundance and nuclear translocation in a time- and concentration-dependent manner

**Figure 1:**
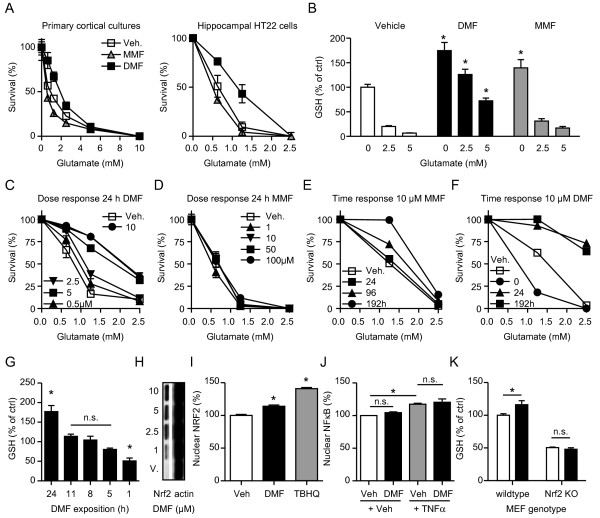
**Dimethyl fumarate (DMF) protects from oxidative stress by enhancing nuclear factor erythroid 2-related factor 2 (Nrf2) abundance and translocation to the nucleus in a time- and concentration-dependent manner.A**) DMF protects from oxidative glutamate toxicity, a model of endogenous oxidative stress where extracellular glutamate blocks the cystine import ultimately causing glutathione (GSH) depletion and cell death. Primary cortical cultures or hippocampal HT22 cells were preincubated with 10 μM DMF, monomethylfumarate (MMF) or vehicle for 24 h and exposed to the indicated concentrations of glutamate for 24 h before cell viability was measured by the Cell Titer Blue (CTB) assay. **B**) DMF and MMF increase cellular GSH concentrations. HT22 cells were treated with 10 μM DMF, MMF or vehicle for 24 h and exposed to the indicated concentrations of glutamate for 8 h before intracellular glutathione was measured enzymatically. **C**-**F**) Dose and time-course of DMF and MMF effect on oxidative glutamate toxicity. HT22 cells were treated for the indicated times and concentrations with DMF, MMF or vehicle before addition of glutamate. Viability was quantitated 24 h later as described above. **G**) Time course of DMF effects on glutathione content. HT22 cells were incubated with 10 μM DMF for the indicated periods of time before intracellular glutathione was measured enzymatically. **H**) DMF enhances Nrf2 abundance quantitated by immunoblots done on nuclear fractions from HT22 cells treated with the indicated concentrations of DMF for 4 h. **I**) and **J**) DMF induces nuclear localization of Nrf2 but has no effect on the nuclear translocation of NF-κB as shown by high content imaging. **I**) HT22 cells were treated with vehicle (n = 9,561 cells), 10 μM DMF for 24 h (n = 8,170 cells) or with 25 μM TBHQ (n = 3,281 cells) as positive control for 4 h. **J**) HT22 cells were treated with vehicle or 10 ng/ml TNFα in the presence or absence of 10 μM DMF (vehicle n = 1,048 cells, DMF n = 943 cells, TNFα n = 1,410 cells, DMF + TNFα n = 1,085 cells). Cells were fixed, stained and nuclear localization analyzed by immunocytochemistry. **K**) DMF has no effect on GSH levels in fibroblasts derived from Nrf2-deficient mice. Cells were treated with 10 μM DMF (black bars) or vehicle (white bars) for 24 h before GSH was measured enzymatically. Graphs of all experiments represent the means ± standard error of the mean (SEM) of three independent experiments performed in triplicate. *P < 0.05, two-way ANOVA with Bonferroni post hoc test in A), B), G), I), J), and paired Student’s *t*-test in K).

Pre-incubation with 10 μM DMF, but not MMF, for 24 h protected primary cortical cultures and hippocampal HT22 cells from oxidative glutamate toxicity (Figure 1A) in line with the increase in cellular glutathione levels seen in both the absence and presence of glutamate (Figure 1B). The protective effect was concentration-dependent for DMF (Figure 1C), but not for MMF at 24 h (Figure 1D). MMF protection took longer to develop and only became evident after incubation for 96 h (Figure 1E), whereas the effect of DMF was already maximal at 24 h (Figure 1F). When added together with glutamate, DMF exacerbated toxicity (Figure 1F) in line with the observation that DMF alone reduced cellular glutathione already after a 1 h exposure (Figure 1G). The transcription factor Nrf2 plays a key role in regulating the expression of proteins involved in GSH metabolism. DMF increased Nrf2 protein abundance at concentrations below 10 μM as shown by immunoblotting of nuclear fractions (Figure 1H) and nuclear localization as shown by immunocytochemistry after 24 h of 10 μM DMF treatment (Figure 1I). The previously reported [17] inhibition of NF-κB translocation to the nucleus by DMF was not evident in these cells. In addition, TNFα, which served as a positive control, induced nuclear translocation of NF-κB, which was not blocked by DMF (Figure 1J). This suggested that the protective effect of DMF in these cells is mainly mediated via the Nrf2-glutathione pathway. In line with this, we observed no increase in glutathione content in response to DMF in fibroblasts lacking Nrf2 (Figure 1K).

### DMF protection involves glutathione recycling

**Figure 2:**
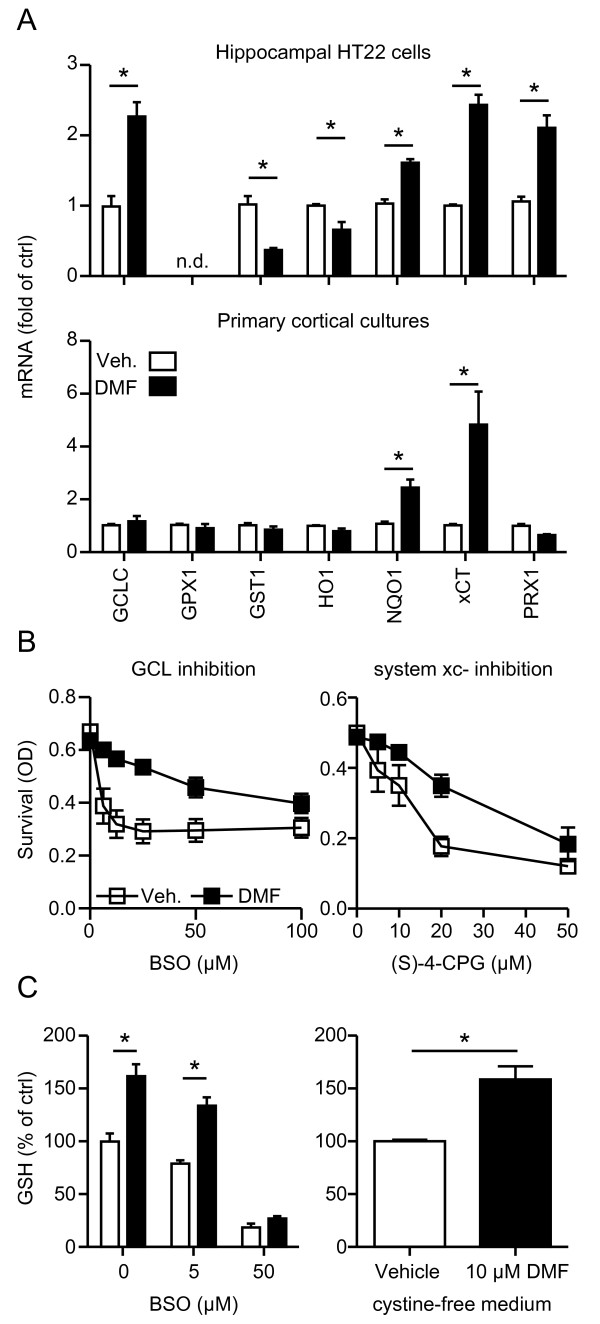
**Dimethyl fumarate (DMF)-mediated protection in neuronal cells involves glutathione recycling.A**) DMF treatment induces mRNA expression of transcripts involved in the antioxidant response. Cells were treated for 24 h with 10 μM DMF or vehicle and mRNA quantitated by real-time PCR using *β-actin* and *hprt* as endogenous controls. **B**) DMF protects from inhibition of GCLC by BSO and inhibition of system Χc − by (S)-4- carboxyphenylglycine (s-4-CPG). HT22 cells were treated for 24 h with 10 μM DMF or vehicle and exposed to the indicated concentrations of s-4-CPG or BSO for another 24 h before cell viability was measured by the CTB assay. **C**) DMF still elevates cellular GSH when GSH synthesis is blocked by GCLC inhibition by BSO or system Χc − inhibition by incubation in cystine-free medium. HT22 cells were treated for 24 h with 10 μM DMF (black bars) or vehicle (white bars) and then exposed to the indicated concentrations of BSO or to cystine-free medium for another 24 or 4 h respectively before intracellular GSH was measured enzymatically. Graphs of all experiments represent the means ± standard error of the mean (SEM) of three independent experiments performed in triplicate.*P < 0.05, paired Student’s *t*-test.

DMF increased the mRNA abundance of various genes involved in the antioxidant response in HT22 cells including the enzymes glutamate-cysteine ligase (GCLC), NQO1, and peroxiredoxin 1, as well as the system Χc- subunit xCT while glutathione S-transferase 1 and heme-oxygenase 1 were downregulated. In primary cortical cultures, only xCT and NQO1 were upregulated by DMF (Figure 2A). We then asked whether inhibition of the function of the most upregulated transcripts, xCT and GCLC with S4-CPG and buthionine sulfoximine (BSO), respectively, abolished the protective activity of DMF. However, DMF was capable of protecting against both compounds (Figure 2B). DMF was also still able to raise glutathione levels when GCLC was inhibited or when system Χc- activity was abrogated by incubation in cysteine-free medium (Figure 2C). Therefore, DMF can still exert protection in neuronal cells when *de novo* glutathione synthesis is blocked, suggesting that it enhances glutathione recycling.

### Neuroprotective concentrations of DMF suppress cytokine production in activated splenocytes from two different mouse strains without exerting effects on viability

**Figure 3:**
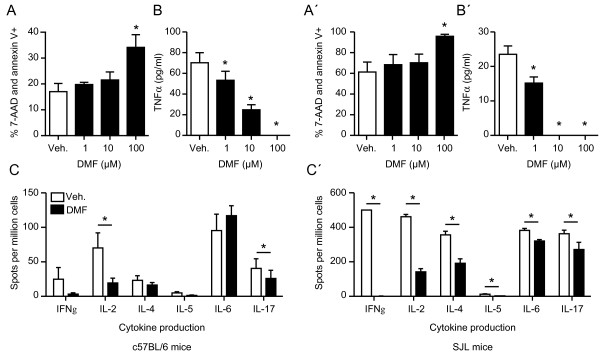
**Neuroprotective concentrations of dimethyl fumarate (DMF) suppress cytokine production in activated splenocytes from two different mouse strains without altering viability.** 10 μM DMF does not significantly affect cell viability of splenocytes from C57BL/6 mice (**A**) or SJL mice (**A’**) while 100 μM is toxic (A) and (A’). Cells were treated for 24 h with the indicated concentrations of DMF or vehicle. Cell viability was measured by flow cytometry quantitating 7-AAD and Annexin V-positive cells. **B**) and **B’**) DMF concentration-dependently reduced TNFα production from anti-CD3-stimulated splenocytes from C57BL/6 (B) and SJL mice (B’). Primary splenocytes from seven mice were treated with the indicated concentrations of DMF for 48 h and co-stimulated with 1 μg/ml anti-CD3 for the same time before TNFα was measured in the supernatants by ELISA. **C**) and **C’**) DMF decreases anti-CD3-induced production of IL-17 and IL-2 in splenocytes from C57BL/6 mice (C) and of the production of IF-γ, IL-2, IL-4, IL-5, IL-6 and IL-17 in splenocytes from SJL mice (C’). Primary splenocytes from seven mice were treated with 10 μM of DMF for 24 h and costimulated with 0.5 μg/ml anti-CD3 for the same time before interferon-gamma, IL-2, IL-4, IL-5, IL-6 and IL-17 were measured using ELISPOT technology. Graphs of all experiments represent the means ± standard error of the mean (SEM) of three independent experiments performed in triplicate.*P < 0.05, paired Student’s *t*-test in (C) and (C’) and two-way analysis of variance (ANOVA) with Bonferroni post hoc test in (A), (A), (B), and (B’).

We then assessed the effects of 10 μM DMF, which was clearly neuroprotective, on the viability and immune response of primary mouse splenocytes isolated from C57BL6 and SJL mice. Apoptosis was quantitated by flow cytometry using Annexin V translocation and cell death by 7-AAD staining. While 100 μM DMF increased dead and apoptotic cells in both strains, the effects of 10 μM were indistinguishable from DMSO, which was used as the vehicle control (Figure 3A for C57BL6 mice and 3A’ for SJL mice). Having established that neuroprotective concentrations of DMF were not harmful to primary splenocytes, we then quantitated TNFα production in response to stimulation with 1 μg/ml anti-CD3 for 48 h. Here, 10 μM DMF attenuated TNFα production in C57BL6-derived splenocytes (Figure 3B) and completely abolished it in SJL-derived splenocytes (Figure 3B’). We also analyzed direct changes in cytokine production in response to anti-CD3 stimulation (0.5 μg/ml for 24 h) with 10 μM DMF or vehicle using ELISPOT technology. This showed a significantly reduced production of IL-2 and IL-17, but no changes in IL-4 and IL-5 in C57BL6 splenocytes (Figure 3C) and a reduction in IL-2, IL-4, IL-5, IL-6, and IL-17 production in SJL splenocytes (Figure 3C’). These data suggest that neuroprotective concentrations of DMF have a prominent immunomodulatory activity, which is more pronounced in the more immune-responsive SJL background without increasing splenocyte apoptosis.

### DMF-treated neuronal cells but not splenocytes secrete neuroprotective GSH

**Figure 4:**
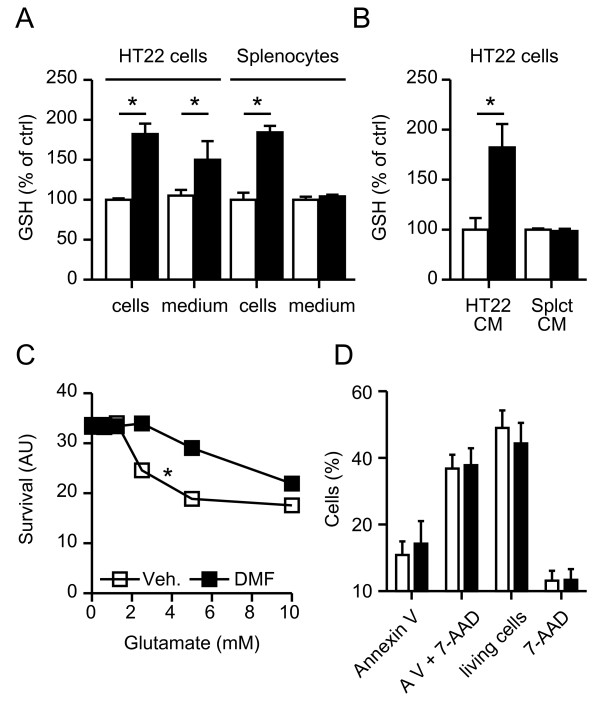
**Dimethyl fumarate (DMF)-treated neuronal cells but not splenocytes secrete neuroprotective glutathione (GSH).A**) HT22 cells and splenocytes show increased intracellular glutathione after 24 h pretreatment with 10 μM DMF, but only HT22 cells release glutathione into the extracellular space. GSH was quantitated enzymatically and normalized to cellular protein content and vehicle-treated cells, respectively. Released GSH in the medium was quantitated after 4 h incubation in cystine-free medium following a 24 h incubation in medium supplemented with 10 μM DMF (black bars) or vehicle (white bars). **B**) HT22 cells treated with conditioned medium (CM) from HT22 cells but not splenocytes take up released GSH and **C**) are protected from glutamate toxicity. HT22 cells were treated for 24 h with conditioned medium before addition of the indicated concentrations of glutamate for another 24 h. Viability was quantitated by the CTB assay. (**D**) Viability of unstimulated splenocytes quantitated by flow cytometry using Annexin V and 7AAD staining is unaffected by HT22 conditioned medium. Graphs of all experiments represent the means ± standard error of the mean (SEM) of three independent experiments performed in triplicate. *P < 0.05, paired Student’s *t*-test for all assays except two-way analysis of variance (ANOVA) with Bonferroni post hoc test for (C).

Pretreatment with 10 μM DMF for 24 h increased the GSH content in both HT22 cells and splenocytes to a similar degree. However, DMF maintained the increase in GSH in cystine-free medium only in HT22 cells but not in splenocytes suggesting differences in GSH secretion or recycling (Figure 4A). We further sought to determine whether these DMF-mediated changes in GSH content and viability of neuronal cells alter the viability of splenocytes and vice versa. HT22 cells and splenocytes were treated with 10 μM DMF or vehicle for 24 h. DMF was then removed and the cells were incubated in DMF-free medium to allow accumulation of secreted GSH (or other DMF-induced secreted substances) for another 24 h followed by transfer of the conditioned medium to untreated cultures for an additional 24 h. The medium conditioned by DMF-pretreated HT22 cells elevated total cellular GSH in new HT22 cells while the medium preconditioned by DMF-pretreated splenocytes did not (Figure 4B). Furthermore, medium preconditioned by DMF-pretreated HT22 cells also protected new HT22 cells from oxidative glutamate toxicity (Figure 4C) but had no effect on the viability of unstimulated splenocytes (Figure 4D). It was not possible to do the reverse experiment using DMF-preconditioned splenocyte medium on HT22 cells, as the oxidative-glutamate-toxicity assay does not work in splenocyte medium.

These data suggest that while both neuronal and immune cells raise intracellular GSH levels upon DMF treatment, only neuronal cells secrete this GSH into the extracellular space where it can protect other neuronal cells. Apparently splenocytes do not secrete GSH and do not benefit from GSH released by neuronal cells, at least under the conditions employed here.

### No effect of DMF on the network activity of primary dissociated cortical cultures grown on multi-electrode arrays

**Figure 5:**
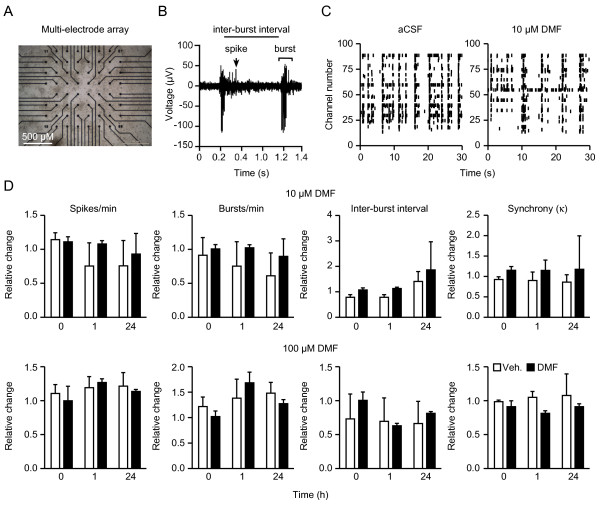
**No effect of dimethyl fumarate (DMF) on the network activity of primary dissociated cortical cultures grown on multi-electrode arrays (MEAs).A**) Dissociated cortical cultures were grown on multi-electrode arrays and visualized by phase-contrast microscopy. Black discs correspond to electrodes. **B**) Typical trace recorded from one MEA electrode showing the parameters used for statistical analysis. **C**) Spike raster plot of spontaneously active cortical neurons on one MEA in artificial cerebrospinal fluid (aCSF) or 10 μM DMF. Each bar represents one spike; electrode numbers are displayed on the vertical axis. The network exhibits correlated burst activity across several electrodes, which is not altered from baseline by DMF. **D**) Treatment with 10 (top) or 100 (bottom) μM DMF for the indicated times does not change the number of spikes and bursts per minute, the inter-burst interval, or the network activity using Cohen’s kappa as measure of synchrony of the activity on all 64 electrodes on the chip. Bar graphs of all experiments represent the means ± standard error of the mean (SEM) of three independent experiments performed in triplicate. *P < 0.05, two-tailed Student’s *t*-test.

MEAs allow a highly sensitive and reproducible assessment of network activity by culturing dissociated cortical cells directly on-chip (Figure 5A). In these cultures, spontaneous electrical activity and signal propagation of evolving neuronal networks can be observed non-invasively in real time at 64 electrodes embedded into the chip. After three weeks *in vitro*, networks exhibit regular bursting activity consisting of single spikes or bursts, which can be measured quantitatively by the spike and burst rate per minute and the inter-burst interval (Figure 5B) [14,18]. Mature neuronal cultures derived from dissociated cortical cells exhibited highly synchronous network activity on MEAs (Figure 5C left panel), but incubation with 10 or 100 μM DMF for 30 sec, 1 h or 24 h did not change the spike or burst frequency, inter-burst interval or synchronicity of these cultures (Figure 5D). We conclude that even excessive concentrations of DMF have no effect on neuronal network activity measured with MEAs.

## Discussion

Our main finding is that DMF at low concentrations protects neuronal cells from oxidative stress by elevating cellular glutathione, and that similar concentrations also reduce production of proinflammatory cytokines from splenocytes. In our experiments, DMF protection needed less time to develop than protection induced by MMF. The induction of the antioxidant response leading to glutathione synthesis seems to be the consequence of an initial and short-lived oxidative stress, since DMF decreased the glutathione content immediately after its addition to the cells. Most likely DMF as an unsaturated carboxylic acid ester initially binds and sequesters glutathione [19]. The long-term effect of DMF in neuronal cells is most probably mediated via Nrf2 as other reported mechanisms such as the inhibition of the nuclear translocation of NF-κB [19] were not evident in these cells and because the increase in GSH synthesis was abolished in cells lacking Nrf2.

On the mRNA level, the most prominently upregulated transcript in HT22 cells and primary cortical cultures was xCT, the functional subunit of system Χc-, which is tightly involved in glutathione homeostasis (reviewed in [13]). DMF, however, also raised the glutathione content when system Χc- activity was inhibited pharmacologically or by incubation in cysteine-free medium, which suggests enhanced glutathione recycling through a mechanism that is as yet unknown.

We observed no upregulation of IL-4 and IL-5 but a significant downregulation of TNFα, IL-2 and IL-17 in DMF-treated anti-CD3-stimulated primary mouse splenocytes from C57BL/6 mice, and additionally IL-4, IL-5, IL-6, IL-17 and IFNγ downregulation in DMF-treated splenocytes from SJL mice, which are a more immune-responsive strain. It has to be kept in mind that in these experiments we analyzed the direct effect of short-term, low-concentration DMF treatment on unsorted splenocytes without priming by antigen-presenting cells. A previous study reported that treatment with 70 μM DMF augmented IL-4 production by CD4+ T lymphocytes *in vitro* only when primed by dendritic cells but not anti-CD3/28 antibodies alone indicating the requirement of antigen-presenting cells for inducing a TH2 response [8,19]. Our data suggest an additional direct effect of DMF on immune cells which is different from its effect during the priming of a T cell response.

Our experiments using media that were preconditioned by DMF-pretreated neuronal cells indicate that both immune and neuronal cells display increased intracellular GSH after DMF treatment but only neuronal cells release this glutathione into the extracellular space where it raises the glutathione content of surrounding neuronal cells and protects them from oxidative stress. The same medium did not prevent unstimulated immune cells from dying which suggests that the death of these cells is not primarily mediated by oxidative stress or that they cannot take up the glutathione. Interestingly, despite elevation of cellular glutathione, DMF-pretreated splenocytes did not release glutathione and their medium did not increase the glutathione content of neuronal cells. As cystine influences the enzymatic glutathione assay employed here by disulfide exchange reactions with glutathione, the measurement of glutathione discharged into the medium was performed in cystine-free medium. Either splenocytes, in contrast to HT22 cells, do not secrete GSH or they have a heightened demand and use up the increased glutathione during the 4 h incubation in cystine-free medium. Alternatively, they might lack the machinery necessary to recycle glutathione.

Treatment with 10 or 100 μM DMF did not alter the activity of primary cortical neurons plated on a multi-electrode array indicating that it has no direct effects on neuronal function *in vitro*.

## Conclusions

In summary, our findings demonstrate that DMF at low concentrations exerts protective effects on neuronal cells and diminishes the production of TNF-α, IL-2, and IL-17 in splenocytes from C57BL/6 mice and the production of all cytokines measured in splenocytes from SJL mice. Although higher concentrations of DMF can cause cell death of primary splenocytes, this is probably not necessary for its immunomodulatory effect. These observations might be relevant for understanding the drug’s presumed mechanism of action as we assume that the active metabolite MMF has similar effects that merely need a longer time to develop.

## Abbreviations

7-AAD, 7-amino-actinomycin D; ANOVA, analysis of variance; BSO, buthionine sulfoximine; CTB, Cell Titer Blue; DMF, dimethyl fumarate; DMSO, dimethyl sulfoxide; ELISA, enzyme-linked immunosorbent assay; GCLC, glutamate-cysteine ligase; GSH, glutathione; IL, Interleukin; IFN-γ, cytokine interferon gamma; MEA, multi-electrode array; MMF, monomethylfumarate; MS, multiple sclerosis; NF-κB, nuclear factor kappa B; Nrf2, erythroid 2-related factor 2; NQO1, NADPH-quinone-oxidoreductase-1; PCR, polymerase chain reaction; S4-CPG, (S)-4- carboxyphenylglycine; SEM, standard error of the mean; TBHQ, tert-butyl hydroquinone; TNFα, tumor necrosis factor alpha.

## Competing interests

This work was supported by an unrestricted research grant by Biogen Idec to AM, and grants from the Deutsche Forschungsgemeinschaft to HHH (Ho 4392/1-1) and the Heinrich Heine University to PA (701220973). HHH was also supported by the Deutsche Multiple Sklerose Gesellschaft and the Strategischer Forschungsfonds der Heinrich Heine Universität. HPH received honoraria with approval by the Rector of Heinrich-Heine University from Bayer Healthcare GmbH, Biogen Idec GmbH, Novartis Pharma GmbH, Teva Sanofi Aventis and Merck Serono GmbH for consulting and speaking at scientific symposia. Other than that the authors declare no competing interests.

## Authors’ contributions

PA and AM conceived the study and participated in its design and coordination, performed the statistical analysis and wrote the manuscript. IB and AI carried out cell viability assays, glutathione measurements, reporter assays, and quantitative real-time PCR experiments. CZ and HHH performed and analyzed the ELISPOT assays. AKM performed the TNFα ELISAs. ZK helped with the reporter assays and cell viability assays. NH and DL performed the translocation assays. JL participated in the study design. PM performed the immunoblotting. NG and KQ performed the MEA analysis. All authors critically revised and approved the final manuscript.

## References

[B1] Compston A Coles A Multiple sclerosis. Lancet 2008 372 1502 1517 10.1016/S0140-6736(08)61620-7 18970977

[B2] Gonsette RE Neurodegeneration in multiple sclerosis: the role of oxidative stress and excitotoxicity J Neurol Sci 2008 274 48 53 10.1016/j.jns.2008.06.029 18684473

[B3] Nave K-A Trapp BD Axon-glial signaling and the glial support of axon function Annu Rev Neurosci 2008 31 535 561 10.1146/annurev.neuro.30.051606.094309 18558866

[B4] Kappos L Gold R Miller DH MacManus DG Havrdova E Limmroth V Polman CH Schmierer K Yoursry TA Yang M Eraksoy M Meluzinova E Rektor I Dawson KT Sandrock AW O’Neill GN bg-12 Phase IIb Study Investigators Efficacy and safety of oral fumarate in patients with relapsing-remitting multiple sclerosis: a multicentre, randomised, double-blind, placebo-controlled phase IIb study Lancet 2008 372 1463 1472 10.1016/S0140-6736(08)61619-0 18970976

[B5] Gold R Kappos L Bar-Or D Arnold D Giovannoni G Selmaj K Yang M Dawson K Clinical efficacy of BG-12, an oral therapy, in relapsing-remitting multiple sclerosis: data from the phase 3 DEFINE trial October 19-22 2011, Amsterdam 17:S9-S52

[B6] Vandermeeren M Janssens S Wouters H Borghmans I Borgers M Beyaert R Geysen J Dimethylfumarate is an inhibitor of cytokine-induced nuclear translocation of NF-kappa B1, but not RelA in normal human dermal fibroblast cells J Invest Dermatol 2001 116 124 130 10.1046/j.1523-1747.2001.00211.x 11168807

[B7] Treumer F Zhu K Gläser R Mrowietz U Dimethylfumarate is a potent inducer of apoptosis in human T cells J Invest Dermatol 2003 121 1383 1388 10.1111/j.1523-1747.2003.12605.x 14675187

[B8] de Jong R Bezemer AC Zomerdijk TP van de Pouw-Kraan T Ottenhoff TH Nibbering PH Selective stimulation of T helper 2 cytokine responses by the anti-psoriasis agent monomethylfumarate Eur J Immunol 1996 26 2067 2074 10.1002/eji.1830260916 8814248

[B9] Linker RA Lee DH Ryan S van Dam AM Conrad R Bista P Zeng W Hronowsky X Buko A Chollate S Ellrichmann G Brück W Dawson K Goelz S Wiese S Scannevin RH Lukashev M Gold R Fumaric acid esters exert neuroprotective effects in neuroinflammation via activation of the Nrf2 antioxidant pathway Brain 2011 134 678 692 10.1093/brain/awq386 21354971

[B10] Lin SX Lisi L Russo Dello C Polak PE Sharp A Weinberg G Kalinin S Feinstein DL The anti-inflammatory effects of dimethyl fumarate in astrocytes involve glutathione and haem oxygenase-1 ASN Neuro 2011 3 75 84 10.1042/AN20100033 PMC3072764 21382015

[B11] Thiessen A Schmidt MM Dringen R Fumaric acid dialkyl esters deprive cultured rat oligodendroglial cells of glutathione and upregulate the expression of heme oxygenase 1 Neurosci Lett 2010 475 56 60 10.1016/j.neulet.2010.03.048 20347008

[B12] Schmidt MM Dringen R Fumaric acid diesters deprive cultured primary astrocytes rapidly of glutathione Neurochem Int 2010 57 460 467 10.1016/j.neuint.2010.01.006 20096739

[B13] Albrecht P Lewerenz J Dittmer S Noack R Maher P Methner A Mechanisms of oxidative glutamate toxicity: the glutamate/cystine antiporter system xc- as a neuroprotective drug target CNS Neurol Disord Drug Targets 2010 9 373 382 20053169 10.2174/187152710791292567

[B14] Steinbeck JA Henke N Opatz J Gruszczynska-Biegala J Schneider L Theiss S Hamacher N Steinfarz B Golz S Brüstle O Kuznicki J Methner A Store-operated calcium entry modulates neuronal network activity in a model of chronic epilepsy Exp Neurol 2011 232 185 194 10.1016/j.expneurol.2011.08.022 21906591

[B15] Lewerenz J Albrecht P Tien M-LT Henke N Karumbayaram S Kornblum HI Wiedau-Pazos M Schubert D Maher P Methner A Induction of Nrf2 and xCT are involved in the action of the neuroprotective antibiotic ceftriaxone in vitro J Neurochem 2009 111 332 343 10.1111/j.1471-4159.2009.06347.x 19694903

[B16] Hofstetter HH Lühder F Toyka KV Gold R IL-17 production by thymocytes upon CD3 stimulation and costimulation with microbial factors Cytokine 2006 34 184 197 10.1016/j.cyto.2006.04.014 16815032

[B17] Loewe R Holnthoner W Gröger M Pillinger M Gruber F Mechtcheriakova D Hofer E Wolff K Petzelbauer P Dimethylfumarate inhibits TNF-induced nuclear entry of NF-kappa B/p65 in human endothelial cells J Immunol 2002 168 4781 4787 11971029 10.4049/jimmunol.168.9.4781

[B18] Gramowski A Jügelt K Stüwe S Schulze R McGregor GP Wartenberg-Demand A Loock J Schröder O Weiss DG Functional screening of traditional antidepressants with primary cortical neuronal networks grown on multielectrode neurochips Eur J Neurosci 2006 24 455 465 10.1111/j.1460-9568.2006.04892.x 16903853

[B19] Ghoreschi K Bruck J Kellerer C Deng C Peng H Rothfuss O Hussain RZ Gocke AR Respa A Glocova I Valtcheva N Alexander E Feil S Feil R Schulze-Osthoff K Rupec RA Lovett-Racke AE Dringen R Racke MK Röcken M Fumarates improve psoriasis and multiple sclerosis by inducing type II dendritic cells J Exp Med 2011 208 2291 2303 10.1084/jem.20100977 21987655 PMC3201195

